# Backward design as a mobile application development strategy

**DOI:** 10.1007/s11423-019-09662-7

**Published:** 2019-04-08

**Authors:** Brinley Kantorski, Camellia W. Sanford-Dolly, Danielle R. Commisso, John A. Pollock

**Affiliations:** 1 Department of Biological Sciences, Bayer School of Natural and Environmental Science, Duquesne University, 600 Forbes Ave., Pittsburgh, PA 15282 USA; 2 The Partnership in Education, Duquesne University, 600 Forbes Ave., Pittsburgh, PA 15282 USA; 3 Rockman et al, Evaluation, Research Consulting, 201 Mission Street, Suite 1302, San Francisco, CA 94105 USA

**Keywords:** Backward design, App development, Science education, Design strategies, Curriculum design methodologies

## Abstract

Backward design is a well-established design strategy that has been used to produce educational curriculum for decades. While traditionally used to plan and create classroom-based curriculum, in this paper we explore the use of backward design as a design strategy for the development of an educational mobile application, *BiblioTech*™ “CityHacks: In Search of Sleep.” We discuss the process from initial conception to launch and updates, as well as plans for future research.

## Introduction

Developed in the late 1990s by Wiggins and McTighe (1998, 2012), backward design is a curriculum design strategy. While backward design may appear to be a logical approach to curriculum design—first identifying what is to be learned, then how to assess that learning, and finally planning instruction—this process is “backward in terms of conventional habits” (Wiggins and McTighe 1998, p. 8). Backward design departs from typical curriculum design practice in several ways. For one, teachers commonly derive learning goals based upon materials easily available to them (e.g., text books, lesson plans, activities), whereas backward design requires teachers to determine learning goals before selecting teaching materials. At the same time, assessment is not considered an integral aspect of curriculum planning, but rather something that is done at the conclusion of instruction. Backward design involves assessment as a key aspect of curricular planning that informs the planning of learning experiences and instruction. In backward design, these two activities—determining learning goals and designing assessment—must be established before learning experiences and instruction can be planned and implemented. McTighe and Wiggins (2012) formalized the backward design process into three consecutive stages, which are as follows: *Stage I* identify desired results, *Stage II* determine acceptable evidence, and *Stage III* plan learning experiences and instruction.

### Design theory

Today, backward design is a well-established curriculum design methodology. We believe that the benefits of backward design can be harnessed beyond traditional curriculum design and applied to the design of apps for educational purposes. Using apps for learning has become an area of great debate among education scholars and practitioners. Even though adoption of digital tablets by schools has become widespread, researchers repeatedly call attention to the lack of evidence-based app development needed to support the multitude of learning apps available (Hirsh-Pasek et al. 2015). It is thought by some that this dearth is the result of the absence of educational researchers involved in the development, design and classroom implementation of tablet apps (Kucirkova 2017). In a study analyzing how iPad apps affect the learning pathways of students, Garry Falloon summarizes the resounding message put forth by many researchers in the field, stating, “Arguments are made for researchers, teachers and developers to work together and adopt methodologies…to radically improve the design of apps used by young students for learning” (Falloon 2013). As both educational researchers and developers of multimedia tools for learning, we realize this immediate need. Based on our assessment, little to no research exists specifically connecting the curriculum design methodology of backward design to app design processes. We feel that our development process for the app *BiblioTech* “Cityhacks: In Search of Sleep” (Pollock and Duquesne University, 2018) may help to address the lack of evidence-based app development strategies in the field of education. This work will also support the educational researchers and pedagogical experts who serve the app development community.

We also find that the design strategy behind the app, *BiblioTech* “Cityhacks: In Search of Sleep” that we have created and its potential use-case scenarios, align closely with the goals and practices of the Open Learning (OL) movement. The OL Initiative promotes collaboration and innovation across disciplines in order to expand traditional classroom learning opportunities (Lewis 1990). More specifically, *BiblioTech* itself was created through the collaborative efforts of science education experts, literacy experts, game designers, neuroscientists, authors, and app developers; all of whom contributed to the design, functionality, and educational content of the app. Furthermore, *BiblioTech* was designed with the intention of varied use-cases appropriate for both traditional classroom and non-traditional OL opportunities such as a flipped classroom, blended learning, or asynchronous learning environment. The adaptive text leveling contained in the app also helps to support the literacy needs of each user as it is perceived by him/her, another important component of OL (Lewis 1992).

The goal of this paper is to show how backward design can be used as an effective guiding strategy for the creation of educational apps. With this study, we hope to add to a growing body of literature that informs future educational app designers of potential strategies and methodologies that can be widely implemented. We feel that the design strategy described here may be of particular interest to educational app developers in that it outlines how to apply the well-established design strategy of backward design to the mobile application development process. Additionally, K-12 educators and instructional designers may find utility in this paper in regards to potential implementation ideas for the *BiblioTech* “Cityhacks: In Search of Sleep” app in both traditional and non-traditional settings.

### “CityHacks: In Search of Sleep”

“CityHacks: In Search of Sleep” is the first edition of *BiblioTech*™, a series of educational apps focused on improving STEM learning, health literacy, and reading skills primarily among young adolescents, but also the general public. *BiblioTech* is a narrative, reading-based app that contains highly interactive features. The platform of *BiblioTech* is a user-centered format that lets users craft their own story by choosing where characters go and what they learn through interacting with a branching, build-your-own adventure style narrative. The app contains a range of features, such as mini-games, infographics, interactive diagrams, video interviews, and a digital notebook. These features all supplement the user’s experience and help them to explore and interact with the story, all while completing a built-in assessment that gauges the user’s understanding of the story content. The text of the app is adaptive in that the user has the ability to choose an appropriate reading level among three possibilities. In “CityHacks: In Search of Sleep,” the app’s goal is to teach users about the importance of sleep and the neuroscience behind it. Additional information about the app can be found on the website of our organization, The Partnership in Education, at https://www.thepartnershipineducation.com/resources/bibliotech-tm-cityhacks-in-search-of-sleep.

### Similarities between curriculum design and app design

As the first installation of the *BiblioTech* series, designing “CityHacks: In Search of Sleep” presented challenges in relation to content, time, and budget concerns similar to those faced during curriculum development. Like teachers or curriculum developers, we were tasked with ensuring that the app effectively taught important and relevant topics (in both science and health) that aligned with existing content standards. At the same time, the app had to be designed in a short period of time. Specifically, our goal was a 1-year timeline to produce, test, and release the app, with plans for its further development to follow. We were challenged to make hard decisions about what information to cover and what to exclude, how best to convey that information, and how to assess learning. The myriad choices for development were limited by cost, in the same way that a teacher is tasked to determine materials and activities based on a classroom materials budget. Overall, the many moving parts foreseen at the earliest stage of development warranted the use of a strategy to identify objectives, guide the project, organize information, and meet learning goals. Therefore, we employed backward design, a research-backed practice that focuses on evidence-based design, to the development of “CityHacks: In Search of Sleep.”

Here we look at the three stages of the Wiggins and McTighe backward design process and demonstrate how they were applied in the design of “CityHacks: In Search of Sleep.” We also briefly examine results of formative and summative evaluation and consider the contribution of this particular backward design process to the app’s demonstrated strengths.

## Methods

### Preliminary conditions

The goal of Stage I of the backward design process is to identify what students should learn in a curriculum. However, before beginning Stage I we determined it was necessary to take a high-level survey of objectives and criteria that we wanted the app to meet. Because the app was developed with support from a Science Education Partnership Award (SEPA) granted by the National Institutes of Health (NIH), the conditions set forth by the agency were identified as guiding tenets. In brief, those conditions translated to: (1) our product must be an innovative educational activity, (2) our product’s intended audience must be Pre K-12th grade teachers and students from underserved communities, and (3) our product must focus on any of the following topics: Courses for Skills Development, Research Experiences, Mentoring Activities, Curriculum, Methods of Development, Informal Science Education Exhibits, and Outreach Activities (NIH 2014). Because of these conditions, our backward design process included more specific constraints than most teachers would normally encounter during curriculum design.

We determined the subject matter of the app would highlight the importance of sleep. This topic was chosen, in particular, due to research conducted on sleep behavior and health in the US at the time, suggesting that inadequate sleep among students impacts mood and behavior, academic performance, and athletic performance. Research suggests that nearly 69% of high school students reported getting insufficient sleep on an average school night, while only about 8% received optimal sleep (Eaton et al. 2010). The Centers for Disease Control and Prevention, CDC (2018), citing over a decade of research, reports children who do not get enough sleep are at a higher risk for several health problems, including obesity, diabetes, poor mental health, and injuries. They are also more likely to have attention and behavior problems, which can lead to poor academic performance. Thus, the need for helping students to establish healthy sleep patterns early in life was apparent at the outset of app development. This need was and continues to be echoed by leading health organizations; sleep was established as a national health priority by Healthy People 2020 (Grandner and Pack 2011), an initiative of the U.S. Department of Health and Human Services, HHS (2010) to improve public health.

Based on research and our team’s background in neuroscience and science education, and through consultation with academic standards, we determined that the app should address the topic of sleep (and why humans need it) and highlight the neuroscience involved in how humans sleep. Therefore, we arrived at a combination of five preliminary conditions that “CityHacks: In Search of Sleep” needed to meet (Table 1).

**Table 1:** Preliminary conditions for “CityHacks: In Search of Sleep” development

Condition numbers	Condition description	Condition types
1	Innovative educational activity	Granting agency
2	Intended for Pre-K to 12th grade teachers and students from underserved communities	Granting agency
3	Focus on any of the following topics: Courses for Skills Development, Research Experiences, Mentoring Activities, Curriculum, Methods of Development, Informal Science Education exhibits, and Outreach Activities	Granting agency
4	Address the topic of sleep and why humans need it	Self-selected
5	Highlight the neuroscience involved in how humans sleep	Self-selected

Aligned with the conditions listed in Table 1, we concluded that the app would: (1) be a fun and engaging educational app that uses an interactive narrative-based format, (2) be intended for use in 4th and 5th grade classrooms and homeschools, as well as the general public, and be able to reach children in underserved communities, including children at risk of developing learning or behavioral disabilities, and (3) focus on skill development, research experience and curriculum related to science, health, and reading proficiency. With these conditions in mind, we turned our attention to conditions 4 and 5 (Table 1), using backward design to identify specific information and learning goals related to sleep.

### Stage I: identify desired results

**Fig. 1:**
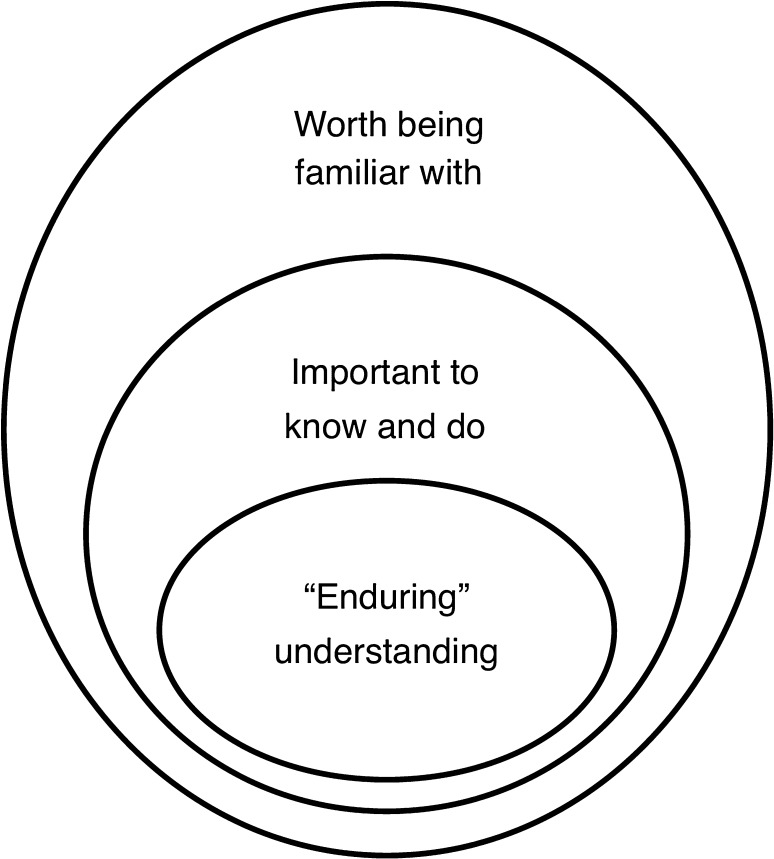
Wiggins and McTighe’s three-layer conceptual model filters information to identify learning goals. Adapted from Wiggins and McTighe (1998, p. 15); with permission)

In the first stage of backward design, Wiggins and McTighe call for identifying what students should “know, understand, and be able to do” (Wiggins and McTighe 1998, p. 9), ultimately equipping learners with the ability to use or transfer what they learn (McTighe and Wiggins 2012). The challenge here lies in establishing priority, or in other words, deciding what content is worth keeping and what content can be discarded, and which content standards are to be addressed. To do this, Wiggins and McTighe (1998) offer a three-layer conceptual model to filter information in order to narrow the focus of content (Fig. 1; Wiggins and McTighe 1998, p. 10). The outermost circle identifies knowledge worth being familiar with; what students should “hear, read, view, research, or otherwise encounter” (Wiggins and McTighe 1998, p. 9). The middle circle identifies what is important to know, including (a) important knowledge, defined as facts, concepts, and principles, and (b) skills, defined as processes, strategies, and methods. The innermost circle identifies enduring understandings, or “big ideas, the important understandings, that [students should] ‘get inside of’ and retain after they’ve forgotten many of the details” (Wiggins and McTighe 1998, p. 10).

McTighe emphasizes the importance of this stage, stating “understanding key concepts and searching for answers to provocative questions—essential questions that human beings perennially ask about the world and themselves—should be the primary goals of teaching and learning” (McTighe and Thomas 2003, p. 52). However, information deemed “important to know” and “worth being familiar with” are part of the narrative that leads to larger understanding, as “factual knowledge and skills are not taught for their own sake, but as a means to larger ends” (McTighe and Wiggins 2012, p. 4). Applied to the development of “CityHacks: In Search of Sleep,” our process followed the general concept model (Fig. 1).

#### Educational standards

Educational standards are the benchmarks employed in K-12 curriculum design. Backward design calls for educational standards to be considered within Stage I, which is a typical starting point for most K-12 teachers. Likewise, because our intention was for the app to be used in 4th and 5th grade classrooms and as part of curricula, our first step in prioritizing the app’s learning goals involved the exploration of existing educational standards; however, our process differed slightly. It was critical to determine a baseline of prior knowledge that users needed in order to use the app. While a K-12 curriculum designer would (ideally) have a good idea of what students have already learned in previous courses, the app was designed for use by both the general public and students in the classroom. Therefore, we needed to make prior knowledge required to use the app minimal, given that prior knowledge levels of the general public are varied and we were not specifically constrained by national or state-specific education standards. However, these standards were still used as a guide during the development process. We elected to create the app with a target reading level and content knowledge equal to that of a 5th grader, using the Next Generation Science Standards, NGSS (2013) to determine what prior science knowledge our target users might have, as well as Flesch–Kincaid Readability metrics (Flesch 1948), Lexile (www.lexile.com), and Dale–Chall tools (1949) for reading skills.

For example, we operated under the assumption that users would be familiar with concepts of behavior patterns, environmental influences, and cause and effect relationships, as these concepts were covered in grades 1, 3, and 4, respectively (NGSS 2013) (Table 2). The text of the narrative contained in the app was written by an established award-winning children’s author, who we requested write the text at a 5th grade level.

**Table 2:** Disciplinary core ideas

NGSS codes	Code description
	Students who demonstrate understanding can:
1-LS1-2	Read texts and use media to determine patterns in behavior of parents and offspring that help offspring survive
3-LS4-4	Make a claim about the merit of a solution to a problem caused when the environment changes and the types of plants and animals that live there may change
3-LS2-1	Construct an argument that some animals form groups that help members survive^a^
3-LS4-3	Construct an argument with evidence that in a particular habitat some organisms can survive well, some survive less well, and some cannot survive at all^a^

Selected from Next Generation Science Standards, NGSS (2013, November). Topic arrangements of the Next Generation Science Standards. Resource document. https://www.nextgenscience.org/sites/default/files/NGSS%20Combined%20Topics%2011.8.13.pdf. Accessed 8 November 2018

^a^Involves “crosscutting concepts: cause and effect relationships are routinely identified and used to explain change” (NGSS 2013, p. 19)

#### Essential questions and enduring understandings

Guided by the educational standards discussed above, we focused in on what information was important to be familiar with, to know, and to understand about sleep, following Wiggins and McTighe’s model (Fig. 1). We arrived at three essential questions users should be able to answer after using the app: (1) Why is sleep important? (2) How many hours should you be sleeping at night? (3) What things can impact our sleep? We determined that if our users were able to answer these essential questions, they would have a good understanding of sleep and its connections to neuroscience. Employing Wiggins and McTighe’s filtering model (Fig. 1), our approach was to rank pieces of information we had gathered about sleep by its ability to answer the essential questions, therefore determining where it fit in the model. For example, users should understand that sleep affects health and performance, know that both humans and animals require certain amounts of sleep, and be familiar with sleep behavior of other species. Through this process, we identified enduring understandings appropriate to our target audience that answer the essential questions:

1. All humans need to sleep. It is an important biological process that allows our bodies time to heal, regenerate, and gain information.
2. Human sleep cycles are controlled by circadian rhythms. Many factors can influence this daily cycle, including: caffeine, food, activity, exposure to light sources, and age.
3. Disrupting sleep patterns can negatively impact human physical and mental health.

These enduring understandings contain answers to the essential questions and provide critical understanding about sleep through both health science and biological contexts, while corresponding to content standards for our target grade levels (Table 3).

**Table 3:** Alignment of essential questions, enduring understandings, and educational standards

Essential questions	Enduring understandings	NGSS standards
Why is sleep important?	1, 3	LS1.A: structure and function
		Plants and animals have both internal and external structures that serve various functions in growth, survival, behavior, and reproduction (4-LS1-1)
How many hours should you be sleeping at night?	1, 2, 3	LS2.A: interdependent relationships in ecosystems
		Organisms, and populations of organisms, are dependent on their environmental interactions both with other living things and with nonliving factors (MS-LS2-1)
What things can impact our sleep?	2	LS1.D: information processing
		Each sense receptor responds to different inputs (electromagnetic, mechanical, chemical), transmitting them as signals that travel along nerve cells to the brain. The signals are then processed in the brain, resulting in immediate behaviors or memories

NGSS derived from Next Generation Science Standards, NGSS (2013, November). Topic arrangements of the Next Generation Science Standards. Resource document. https://www.nextgenscience.org/sites/default/files/NGSS%20Combined%20Topics%2011.8.13.pdf. Accessed 8 November 2018

Wiggins and McTighe (1998) suggest that enduring understanding should, in summation, (1) focus on larger concepts, principles, or processes that will typically be more difficult for students to grasp and will harbor misconception, (2) have application value beyond the classroom and into the future, and (3) engage students and sustain inquiry. Our three enduring understandings meet this criteria. First, they are overarching critical concepts about sleep that work in concert together and are necessary to develop a greater understanding of the importance of sleep, and more broadly, of science and health. Simultaneously, they are concepts that tend to be difficult for students to grasp; misconceptions about sleep abound, as most don’t fully understand the consequences of lack of sleep, nor the physiology behind its physical and mental effects. Second, our enduring understandings have application value beyond the classroom. Backward design stresses the importance of considering knowledge transfer at this stage, with the goal being that students should effectively be able to transfer knowledge and skills to new and different contexts as a marker of true learning (McTighe and Wiggins 2012). The identified enduring understandings about sleep can be directly applied to a person’s life to improve their health and can provide a basis of understanding of scientific principles to benefit science education outcomes for students. Third, our enduring understandings hold the capacity to engage students and sustain inquiry. Considering that sleep is a deep and complex topic and an area that current scientific research is still exploring, there is no shortage of questions about sleep that are still being investigated.

If we accomplished nothing else with our app, our goal was to convey these three enduring understandings in a meaningful and accessible way to our users. These facts fit into the larger goals of our granting agency, the NIH, who states its mission is to “seek fundamental knowledge about the nature and behavior of living systems and the application of that knowledge to enhance health, lengthen life, and reduce illness and disability” (NIH 2018). By educating our users on the importance of sleep we are helping to create a more well-informed and health-conscious population. Having identified our enduring understandings the app would convey, we then turned our attention to the second stage of backward design, focusing on assessment.

### Stage II: determine acceptable evidence

**Fig. 2:**
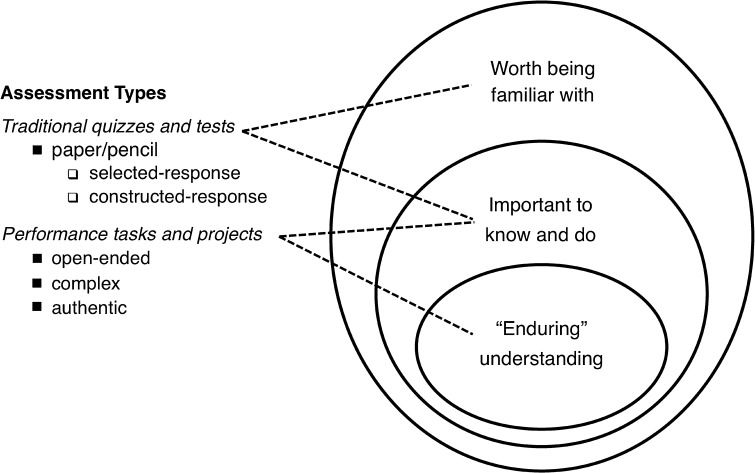
Determining types of assessment through backward design (Wiggins and McTighe 1998, p. 15; with permission)

The main thrust of Stage II of the backward design model is to “encourag[e] teachers and curriculum planners to first think like an assessor before designing specific lessons and units,” which requires considering how to determine whether or not students obtain understandings at the outset of design (Wiggins and McTighe 1998, p. 12). It is necessary for designers to determine what counts as acceptable evidence for meeting desired learning goals before embarking on the final stage of backward design—planning learning experiences and instruction. To guide evaluation in backward design, a variety of assessment types and outcomes can be employed, emphasizing that enduring understanding should be probed through “performance tasks or projects” which are “open-ended, complex, and authentic” (Wiggins and McTighe 1998, p. 15), as seen in Fig. 2.

Performance tasks, such as a long-term project, should be the culmination of learning that explores true understanding. However, traditional types of assessment of other information, such as quizzes and tests, “round out assessment” and ensure that key information is being accounted for (McTighe and Wiggins 2012, p. 13).

#### Assessment

In order to determine if the app’s users had gained the knowledge we set forth to convey (namely the three key concepts on sleep), we created a cumulative in-app assessment tool. Ultimately, this tool would test the culmination of learning and determine if the enduring understandings were truly gained by the user, which would in turn facilitate self-reflection on learning for both classroom and general public users. To accomplish this, we designed the in-app assessment tool as a functional aspect of the app that is built into the narrative of the story itself. For brevity, the narrative is summarized below:

The user plays through the story in the app as a teenage girl, Maya, who wants to attend a coding club which meets from 8PM-12AM on Thursdays and Friday. Her mother does not want her staying up late on a school night, so she says that unless Maya can convince her otherwise, she will not be permitted to go to the coding club meetings. As Maya, the player has autonomy in the narrative to choose what locations she can go to in order to learn more about sleep using a branched build-your-own-adventure format. As the story progresses, the player as Maya, visits locations and collects facts and information about sleep in their digital notebook and returns home to try to convince Mom to let her stay up late by pulling facts out of the user’s digital notebook.

**Fig. 3:**
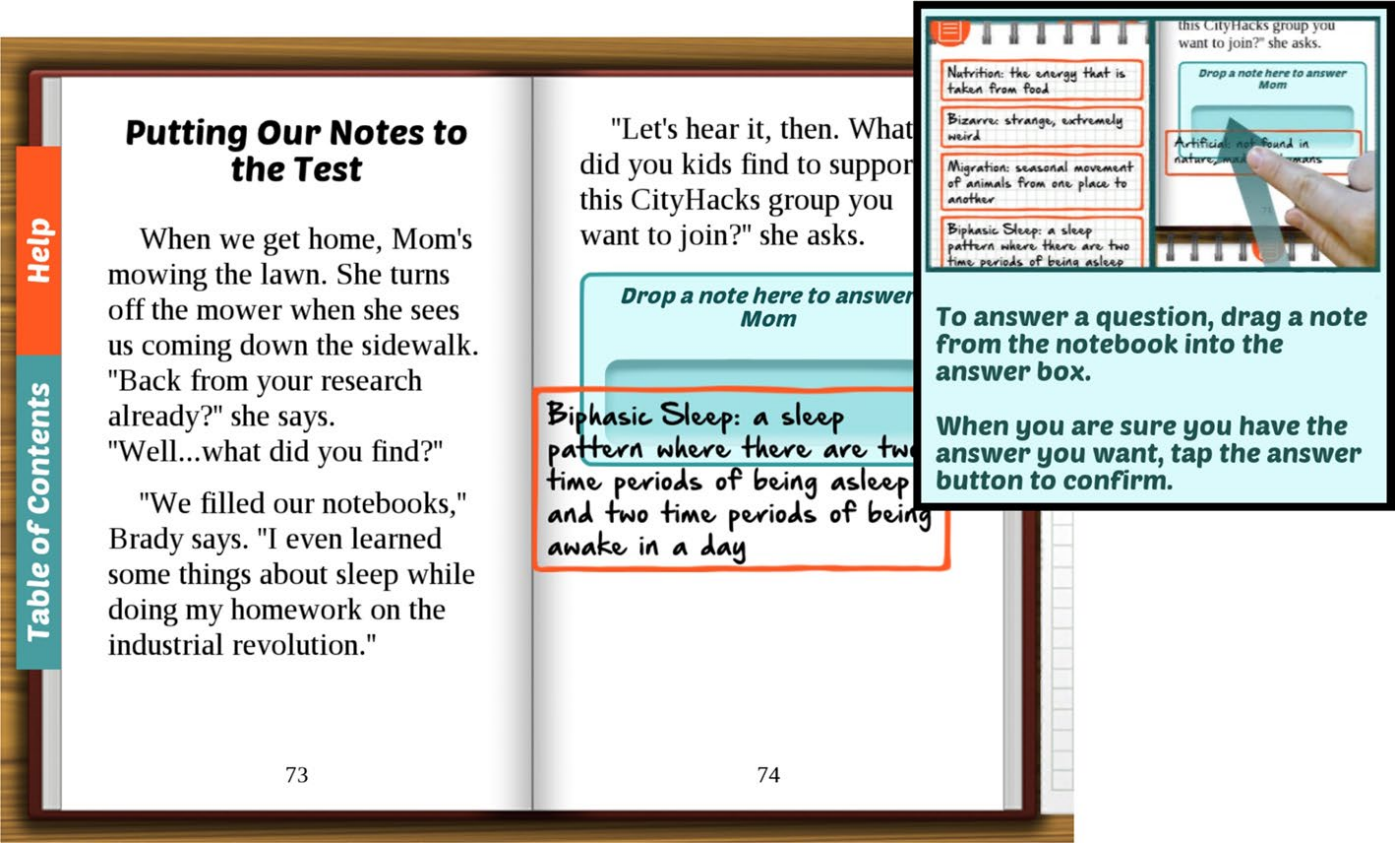
A screenshot of the debate assessment where users must use specific sleep facts to convince Mom to let Maya and her brother Brady attend the coding club; inset shows a page from the tutorial illustrating how to drag notes to answer Mom’s questions

To win the debate and convince “Mom,” Maya must argue that staying up late once in a while is not terribly detrimental but that kids should not make a habit of staying up late all the time, using key pieces of information, or specific sleep facts, that support Maya’s argument (Fig. 3). These specific sleep facts include:

1. In the past, before the industrial revolution, humans had biphasic sleep patterns.
2. There are no long term negative effects to staying up late once in a while, but being chronically sleep deprived has very negative effects on your physical and mental health.
3. Napping can help you to catch up on missed sleep only if it is done within 24 hours of missing sleep.
4. Exposure to artificial light and caffeine can disrupt a person’s normal sleep cycle.

**Fig. 4:**
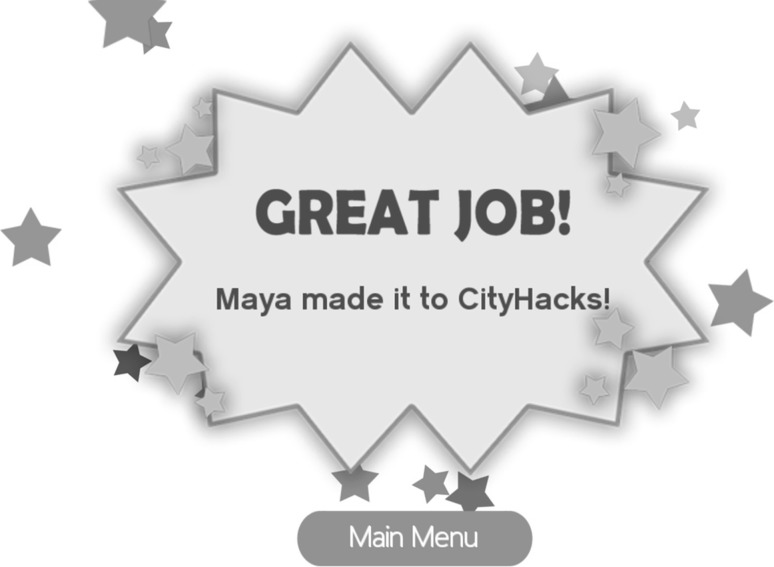
A screenshot from the successful “pass” ending, where the user has convinced Mom to let Maya stay up late and attend the coding club

If the user supplies the correct sleep facts, they pass the assessment and Maya is allowed to attend the coding club meetings. If the user does not provide the correct facts, Maya fails and is not permitted to attend the coding club meetings. However, if Maya fails on the first attempt, she is given another chance to revisit some locations in the story to look for more supporting facts, and user choices are then limited only to locations where relevant sleep facts are found. This scaffolded process helps users who may need more repetition of the content for mastery. After this, the user is allowed to re-attempt the debate assessment and can pass or fail depending on the facts given during the debate. Whether the user passes or fails, the story ending is affected and users can easily observe and understand if they were successful or unsuccessful in the assessment (Fig. 4).

McTighe and Wiggins (2012) suggest that when someone truly understands information, they can, in brief: (1) explain concepts, principles and processes, (2) interpret information in various ways, (3) apply knowledge to new and complex contexts, (4) demonstrate perspective, (5) display empathy, and (6) display self-knowledge. However, they note that not all six facets can or should necessarily be used in assessment, and only those that provide appropriate evidence of the targeted understanding should be considered.

In “CityHacks: In Search of Sleep,” users decide what information to collect as they read (interpret information), where to go in order to gather information, and what information to use to support their argument (explain concepts and apply knowledge to new/complex contexts). As they read and take notes, they are also learning and refining their argument. For example, Maya learns about the negative effects of chronic sleep deprivation on health, causing her to rethink her case for staying up late.

Overall, the app does not rely solely on testing for facts or choosing answers in multiple choice style. In a study on a novel mobile device software for learning, researchers Fuad et al. (2018) point out the inadequacy of multiple choice and true/false assessment that are commonly associated with using mobile devices in classrooms. They observe that despite providing immediate evidence of learning, these assessment techniques do not test for a student’s true comprehension and skill mastery, and are especially inadequate when it comes to interactive problem-solving skills that students should acquire in STEM courses. In “CityHacks: In Search of Sleep,” the interactive narrative format and notebook allows for greater assessment of the user’s problem-solving skills, as well as reading and comprehension, where standard multiple choice and true/false techniques would fall short of accurately measuring mastery. Overall, the interactive narrative functions as a complex, authentic performance task that contextualizes learning by requiring users to make key decisions that affect the story’s outcome.

While using the app, users encounter simple mini-games, puzzles, and activities interspersed throughout the story, such as crossword puzzles, which do not affect the story’s overall outcome. Each of these activities uses words and concepts from the relevant passages that have been read. While helping to break up long passages of text and keep readers engaged, the mini-games and activities let users self-assess and further build their familiarity with and knowledge of key vocabulary words in the story. Aligning with McTighe and Wiggins’ assessment types (Fig. 2), these mini-games function similarly to traditional quizzes and overall contribute to learning enduring understandings. For example, users familiarize themselves with the word “monophasic” and learn its meaning in both the text, in infographics and in mini-games, better helping them to understand how circadian rhythms that control sleep can become disrupted.

### Stage III: plan learning experiences and instruction

In this final stage of the backward design framework, curriculum designers have prioritized information and identified evidence for understanding. According to Wiggins and McTighe (1998), the curriculum designers are now in an optimal place to plan instruction to meet their learning goals. Instructors are tasked to consider key questions to meet desired goals, surrounding how best to teach, what activities to do, what materials and resources to use, and if their design thus far is coherent and effective.

At this point we had identified enduring understandings and employed an organizational schema for information, as discussed in Stage I, and developed methods for learning assessment; now we were challenged to determine the vehicles that would best deliver that information for learning, such as mini-games, video interviews, infographics, or the story text itself. Similar to the way that teachers and curriculum designers would apply knowledge of learning and best teaching practices in devising and implementing instructional activities, we employed knowledge, research, and skills in education and multimedia design. We also included formative and summative evaluation, to build out the interactive features of the app. Special attention was paid to at-risk learners in underserved communities and those with learning disabilities. Below we highlight ways in which this informed design.

#### Differentiation for diverse learners

In designing the app, considerations about the range of reading abilities and access to technology were discussed. Because the app is available to the general public, we realized that our users would possess a wide range of reading abilities. It is estimated that 37% of US 4th-graders fail to obtain basic levels of reading achievement, and cannot successfully complete schoolwork. That rate is more pronounced among low-income families and ethnic minority groups; 56% of Latino and 60% of African American students read at below-basic levels (National Early Literacy Panel 2008). Likewise, research from the National Center for Education Statistics shows that when tested, 18% of US adults (about 50 million people) performed at the lowest level of literacy scale, as measured by the international PIAAC literacy scale (NCES n.d.). Therefore, we needed to consider users who would not be able to effectively read the app’s content. As such, a new feature which allows users to choose the reading level of the story was developed.

Upon analyzing the finished narrative text, we found that the text had a Flesch–Kincaid grade level score of 4.8, implying that the text would be able to be read and understood by someone who had completed the majority of 4^th^ grade and was entering 5^th^ grade, which matched our target audience grade range. To expand the utility and accessibility of the app to more students of different abilities we invented the Adaptive Reader™ technology. This feature converts the reading level of the text from a story suitable for 5^th^ grade level to either a 3^rd^/4^th^ grade level or up to an 8^th^ grade level, depending on the option chosen by the user.

Recognizing that the Flesch–Kincaid and Lexile tools measure text complexity very similarly—sentence- and word-length—but Lexile scores are the universally recognized leveling tool in K-12 education, we also used Lexiles to measures the text. We analyzed the app’s text section-by-section to maintain a high level of consistency across the three reading levels; this made sure that each section was leveled one tier up and one tier down from the medium-level text. While there is no semantic component to the Lexile score, given the heavy scientific and technical load of the app’s content, we also used the New Dale–Chall Method (1995), which is based on vocabulary, as well as the EDL Core Vocabularies book (Taylor et al. 1989). Simply lowering the Lexile level to 3rd–4th grade would not have changed the true readability of the text (because of the challenging concepts and vocabulary).

**Fig. 5:**
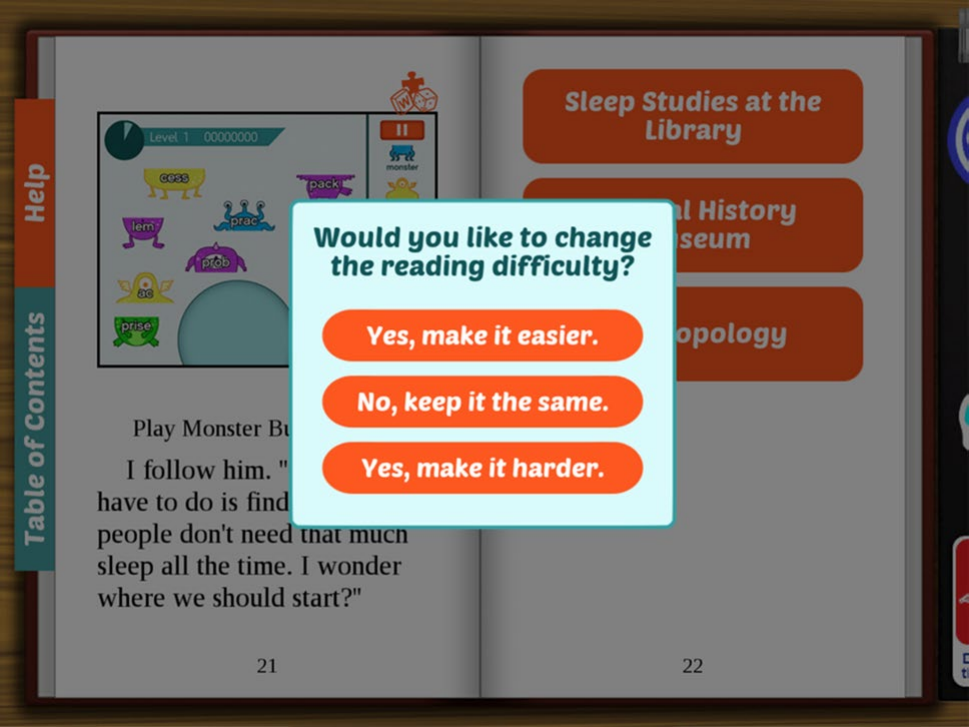
A screenshot of the proposed feature that will allow users to choose the reading level they feel most comfortable using

Figure 5 shows an example of how the Adaptive Reader technology works. A pop-up box appears at the end of selected chapters of the story, where the user has the option to continue at the same reading level or choose an easier or more difficult level. This feature allows the text to change based on the needs of the individual user. While the reading difficulty adapts, the scientific content that is provided to each user stays the same. In this way, peers each read at their appropriate reading level but are still be able to access the same content and participate in discussions without feeling left out or excluded from full participation because of their reading skills.

Currently, “CityHacks: In Search of Sleep” includes Adaptive Reader technology (with three reading levels) and is available for iOS and Android devices. Adaptive text is one of the app’s features that allow the content of the app to be accessed by a diverse audience of learners.

#### Evaluation

Formative evaluation provides developers with iterative feedback to improve product design, while summative evaluation investigates whether a project’s overall goals and learning objectives were met (Scriven 1996). An independent educational research and evaluation firm, Rockman et al (http://rockman.com/), conducted formative assessment of “CityHacks: In Search of Sleep” with two different audiences: 5th grade students in the classroom (N = 34; 15 females, 19 males) and adult literacy learners at the Greater Pittsburgh Literacy Council for GED and English language learners (N = 7) via a focus group methodology (Morgan 1997). In these focus groups, participants were shown an early version of the app, and were asked for their opinions on the story narrative, the proposed mini-games and interactive diagrams, the digital notebook feature, as well as the interface design and readability of the text. Focus groups with adult literacy learners revealed that both older and younger potential app users were interested in reading about health topics, which suggested broader appeal for the app itself. Participants shared that they did not often take notes while reading because they did not want to go back and forth between paper and a device—thus, “CityHacks: In Search of Sleep’s” built-in note-taking feature was of interest to them. Furthermore, adult literacy learners struggled with word definitions and reading comprehension, issues that were addressed in the final design of “CityHacks: In Search of Sleep” via clickable terms and the ability to adjust the reading level up or down.

Focus groups with two 5th grade classes (7 females, 9 males) and (8 girls, 10 boys) indicated that youth using the app most liked the ability to select locations that they visited in the story (“I liked how you could choose how Maya got information”; “I really liked how you could create your own story while also researching information on the brain”). In one of the classes, all but one student in the focus groups (N = 16) felt that they had learned something from the app: “I learned information that I didn’t know before and it was told to me in a fun way.”

The data from the formative evaluation was used during the design phase to inform the decision-making process; many participant ideas were incorporated into the current version of the app. In addition, these formative evaluations also helped us to better understand what our potential users wanted and needed in an app and thus, helped to further inform the development process.

**Fig. 6:**
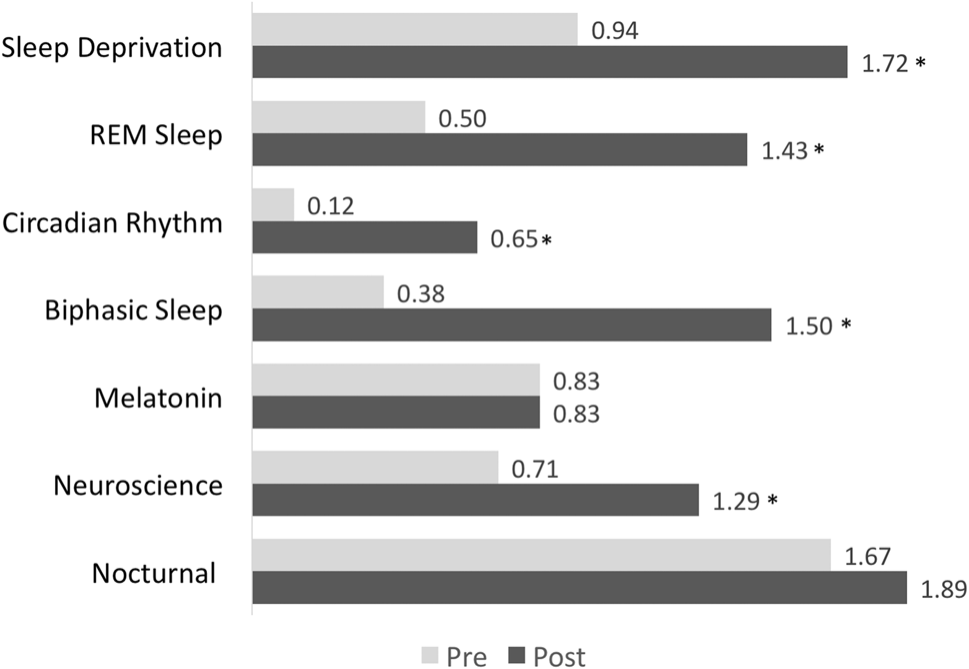
Students’ familiarity with sleep-related vocabulary before and after reading “CityHacks”. A 3-point scale with 0 = "Never heard of it," 1 = "Heard of it, but don't know what that is," and 2 = "Know it." *Indicates a significant difference at the p < .05 level

**Fig. 7:**
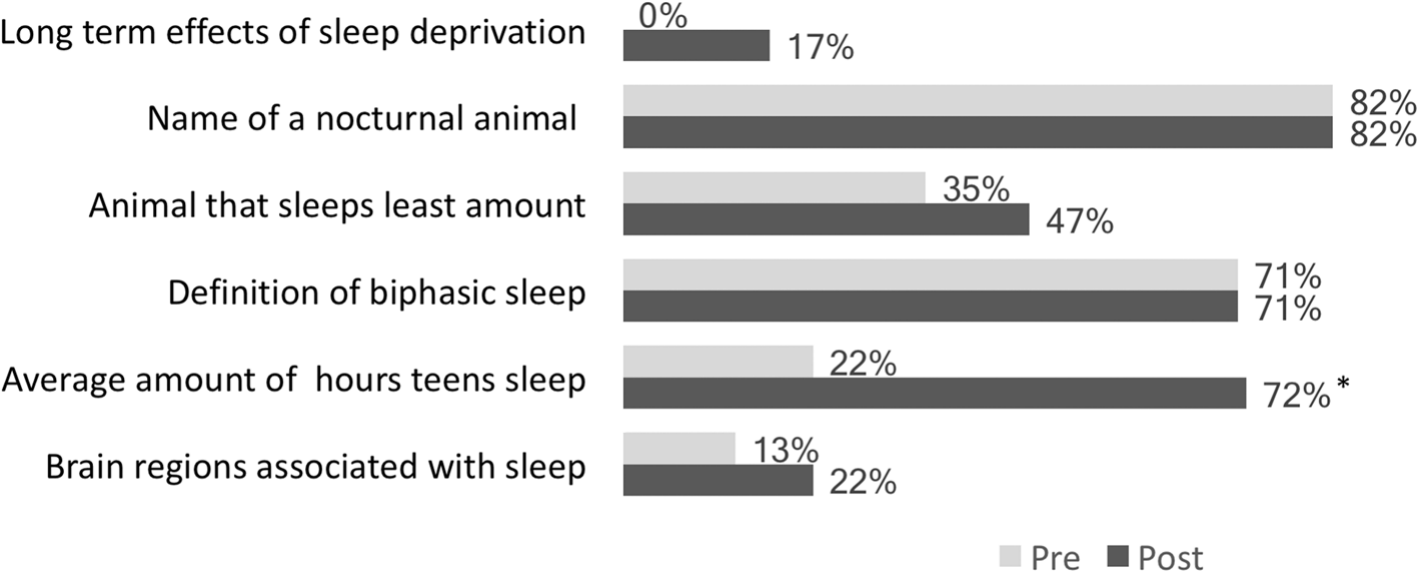
Percentage of correct responses to sleep-related content questions before and after reading “CityHacks”. * Indicates a significant difference at the p < .05 level.

Summative assessment on earlier versions of the app were conducted, again, with 5th grade students (N = 18) in a classroom setting. In this case, pre–post surveys were distributed to the students of the class that asked specific questions about science content related to sleep. Some of these survey prompts included the essential questions listed earlier. Findings from these pre–post surveys show that student understanding related to the topic of sleep increased after using the app (Figs. 6, 7).

For example, use of the app impacted students’ familiarity with sleep-related terminology (Fig. 6) and overall sleep-related content knowledge (Fig. 7). Here, students significantly improved their understanding of key sleep-related topics (as measured by their correct responses on 10 multiple choice questions) from pre (M = 3.64 out of 10) to post (M = 4.75 out of 10); t(13) = − 3.143, p = .008. In addition, students’ responses to three open-ended questions about the neuroscience of sleep were coded to see if there were any changes in their answers from pre-to-post. After using the app, students were (1) significantly more likely to be able to identify factors that can affects one’s quality of sleep, (2) better able to identify some of the effects of sleep deprivation, although this result was not significant, and (3) significantly better at identifying the benefits of a later school start time for teenagers (Table 4). Taken together, these results demonstrate that the app met its learning objectives for its target audience by supporting a deeper understanding of key concepts and ideas that answer the essential questions (i.e., “enduring understandings” and “what is important to know and do”), as well as terminology related to those questions that is “worth being familiar with” (i.e., details that contribute to a learner’s deeper understanding).

**Table 4:** Demonstrated improved understanding of the neuroscience of sleep from pre to post testing

Learning goals	Percentage of students who answered correctly on the pre-test	Percentage of students who answered correctly on the post-test	*p* values
Able to identify…			
Factors that can affect one’s quality of sleep	17	61	.002*
Effects of sleep deprivation	11	39	.056
Benefits of a later school start time for teenagers	11	33	.042*

*Significant difference at the p < .05 level

#### Implementation and refinement

In a traditional backward design model, the implementation portion would include the delivery of the instruction in a classroom. In our case, however, implementation is a bit different. “CityHacks: In Search of Sleep” is currently available as a free download for iOS and Android devices from their respective app stores and can be used on virtually all smartphones and tablets. Recent research (Anderson 2017) suggesting that lower-income Americans are more likely to rely on smartphones for internet access than they are on computers or tablets influenced our decision to launch “CityHacks: In Search of Sleep” as a free mobile app, instead of a browser-based or desktop software package.

Future goals include implementing and testing a built-in stealth assessment tool, which will function as an informal metrics-based assessment allowing teachers to track and monitor student progress as they advance through the app. Data from students’ gameplay, such as the debate assessment, embedded mini-games, and reading behaviors (i.e., words per minute and selected reading level, among other parameters) will be sent to the assessment dashboard where the teacher can view it in various forms. In the near future, we will begin defining specific ranges and scores for each category of evidence that will indicate if learning has occurred. Our goal with the design and functionality of this stealth assessment feature is to eliminate the need for additional outside assessments to be administered, which require additional class time for administration and grading.

## Discussion

Before we began the development of what has become the *BiblioTech* platform, we established a theoretical framework based on a recursive logic model. The logic model considers the inputs of producers and the science content, as well as curriculum and learning goals. Next, active formative assessment, preliminary research and beta-testing of activities guide the development of the user experience. Then, the completed app is subjected to user testing as described here. The creation of *BiblioTech* “Cityhacks: In Search of Sleep” was thus, the result of a highly collaborative effort of many educational stakeholders: science education experts, literacy experts, game designers, neuroscientists, authors, and app developers, all of whom worked toward the common goal of creating an educational tool that can help learners both inside and outside of the traditional classroom. Through the use of Wiggins and McTighe’s backward design framework, and informed by OL design concepts, we were able to map out the app development process to assure that the design choices being made were in alignment with our identified goals and supported by the recursive testing and feedback defined by our model. The inclusion of adaptable text leveling in the app was a conscious effort to allow for student choice and autonomy in learning goals. Taken together, these components allowed for the creation of an educational tool that can be easily integrated into an existing K-12 curriculum or utilized in more non-traditional educational settings. As a platform, *BiblioTech* adaptive readers can support a wide variety of narratives with diverse content and learning goals.

### Design challenges

Simply stated, the challenge was to design an app that could leverage the educational benefits afforded by a mobile application and avoid making design mistakes that would impede learning. All of this had to be done in a time-sensitive manner and, of course, on a set budget. Despite the large amount of learning apps available, research offered few iPad and tablet studies to go on at the start of production in 2014, so our development involved a high degree of experimentation. While we determined backward design could be an effective development framework for tablet learning, other research needed to be considered to avoid the hazards inherent in multimedia design. In *Multimedia Learning*, Clark and Feldon analyze 10 “questionable principles,” or widely-held and practiced beliefs regarding multimedia learning, pointing out that multimedia designers who use new technologies for learning, such as the iPad, without research-based knowledge of the shortcomings embedded in these questionable principles, run the risk of failure (Clark and Feldon 2014).

One significant “questionable principle” we addressed in “CityHacks: In Search of Sleep” was that student-managed constructivist and discovery approaches were beneficial to learning. Research indicates that not all students benefit from navigating unstructured learning environments with little instructional support. In fact, it’s suggested that students with higher degrees of prior knowledge learn best with minimal instruction, whereas those with low to moderate levels or prior knowledge learn best with more guidance (Clark and Feldon 2014). Thus, it was important that we establish prior knowledge levels, discussed earlier. Still, we were challenged to determine an appropriate level of guidance that could benefit users of various backgrounds.

User-testing proved to be an integral key toward reaching our final outcome. Originally, the digital notebook—a place for the user to store relevant facts and information that they gather throughout reading the story—was designed to allow for free-form user input via the keyboard. However, in very early focus group testing, we found that many users struggled to understand or differentiate what information needed to be remembered or marked as important. Many expressed frustration with the notebook and were unable to identify what information needed to be stored and how to summarize that information in a meaningful way. Based on this feedback, we changed the design of the notebook to eliminate free-form text input and to only allow drag-and-drop style information gathering. By scaffolding the note-taking process (Lajoie 2014), users responded much more positively—their frustration levels were reduced and they were able to focus and engage with the story more effectively.

**Fig. 8:**
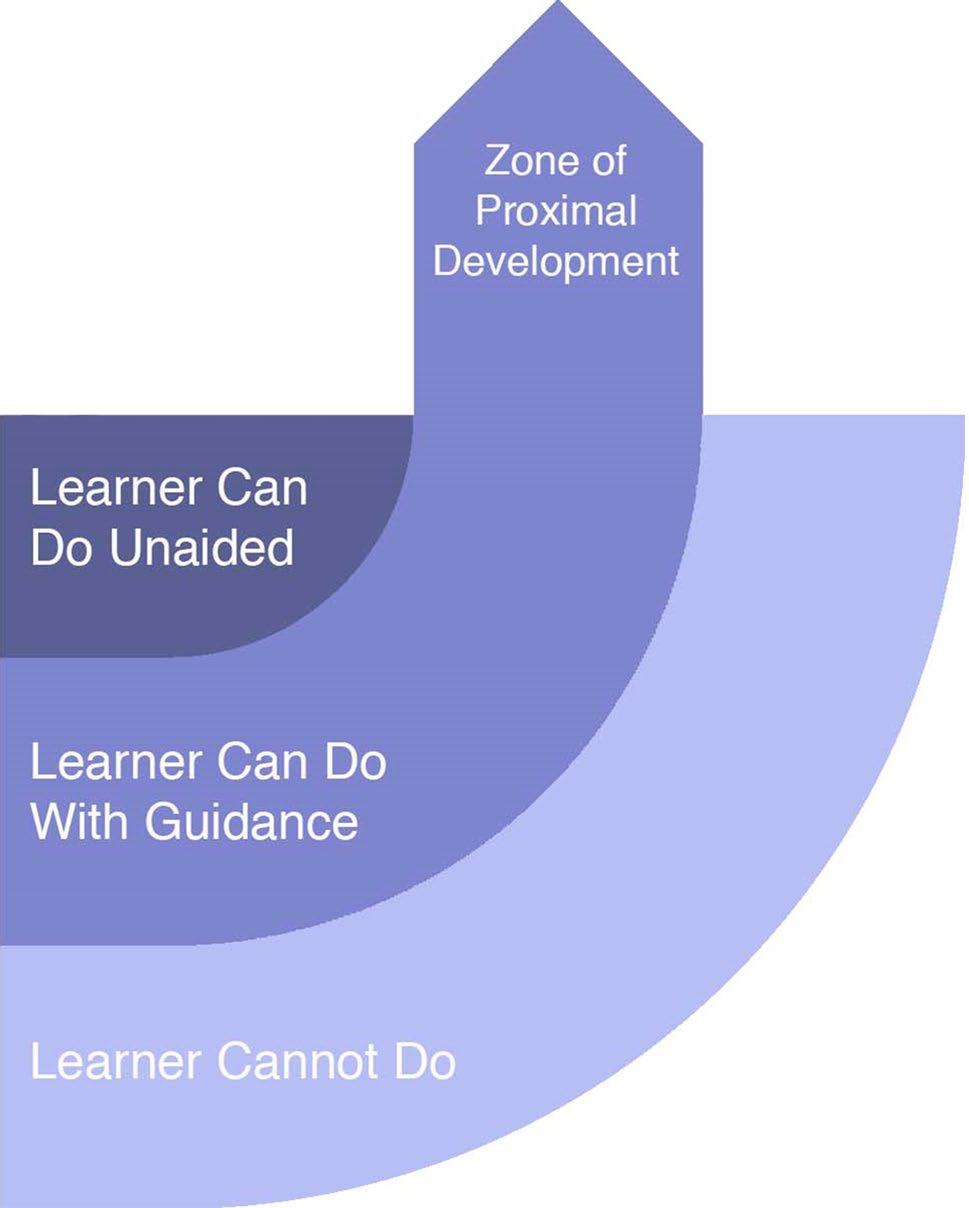
The Zone of Proximal Development illustrates gaps between guided and non-guided learning

This heightened level of guidance of reading-based learning corresponds to Lev Vygotsky’s theory of the zone of proximal development, bridging the gap between what a learner is able to accomplish without help and what a learner is not able to accomplish (Podolskiy 2012) (Fig. 8).

With instructional scaffolding, summarized as a “pedagogical process experts use to help learners perform tasks they cannot do by themselves” (Lajoie 2014, p. 628) users progressed into the zone of proximal development where they were able to complete the note-taking task with the help of drag-and-drop information items. Our goal is that “Cityhacks: In Search of Sleep” provides the guidance that lands users firmly in their zone of proximal development, and we continue to test and refine the app in order to achieve this balance.

## Conclusion

In sum, the three-stage Wiggins and McTighe’s backward design process provided a systematic conceptual framework for educational app design that tied together the identification and implementation of learning goals and assessment goals with the design of the app itself. Ultimately, it lent a comprehensive direction to the design process of “CityHacks: In Search of Sleep,” and findings from early evaluation studies showed the app to be effective in allowing students to successfully complete the identified learning goals. Broadly, this suggests applying a backward design framework for developing learning apps may be beneficial, but to a certain extent—design should also involve evaluation and the application of other relevant research theories and practices to best leverage the strengths of the technology to provide effective learning.
